# Spatial Heterogeneity of Soil Nutrients after the Establishment of *Caragana intermedia* Plantation on Sand Dunes in Alpine Sandy Land of the Tibet Plateau

**DOI:** 10.1371/journal.pone.0124456

**Published:** 2015-05-06

**Authors:** Qingxue Li, Zhiqing Jia, Yajuan Zhu, Yongsheng Wang, Hong Li, Defu Yang, Xuebin Zhao

**Affiliations:** 1 Institute of Desertification Studies, Chinese Academy of Forestry, Beijing, 100091, China; 2 Research Institute of Forestry, Chinese Academy of Forestry, Beijing 100091, China; 3 Sand Control Experimental Station of Qinghai Province, Gonghe 813005, Qinghai, China; University of A Coruña, SPAIN

## Abstract

The Gonghe Basin region of the Tibet Plateau is severely affected by desertification. Compared with other desertified land, the main features of this region is windy, cold and short growing season, resulting in relatively difficult for vegetation restoration. In this harsh environment, identification the spatial distribution of soil nutrients and analysis its impact factors after vegetation establishment will be helpful for understanding the ecological relationship between soil and environment. Therefore, in this study, the 12-year-old *C*. *intermedia *plantation on sand dunes was selected as the experimental site. Soil samples were collected under and between shrubs on the windward slopes, dune tops and leeward slopes with different soil depth. Then analyzed soil organic matter (SOM), total nitrogen (TN), total phosphorus (TP), total potassium (TK), available nitrogen (AN), available phosphorus (AP) and available potassium (AK). The results showed that the spatial heterogeneity of soil nutrients was existed in *C*. *intermedia* plantation on sand dunes. (1) Depth was the most important impact factor, soil nutrients were decreased with greater soil depth. One of the possible reasons is that windblown fine materials and litters were accumulated on surface soil, when they were decomposed, more nutrients were aggregated on surface soil. (2) Topography also affected the distribution of soil nutrients, more soil nutrients distributed on windward slopes. The herbaceous coverage were higher and *C*. *intermedia* ground diameter were larger on windward slopes, both of them probably related to the high soil nutrients level for windward slopes. (3) Soil “fertile islands” were formed, and the “fertile islands” were more marked on lower soil nutrients level topography positions, while it decreased towards higher soil nutrients level topography positions. The enrichment ratio (E) for TN and AN were higher than other nutrients, most likely because *C*. *intermedia *is a leguminous shrub.

## Introduction

Desertification is one of the main types of land degradation in arid, semi-arid and dry sub-humid areas and results from various factors, including climatic variability and human activities [1]. Land desertification affects 25% of the total land area on Earth, impacting over 250 million people [2]. Sand mobility and wind erosion result in coarse, poor soil and low land productivity, which can degrade the human living environment and impede socioeconomic development. As China has been severely affected by desertification, desertification control is considered a principal strategy for maintaining ecological security in northern China. Vegetation restoration is a common and effective way to combat and control desertification [3], and it has undoubtedly contributed to an improvement in environmental conditions, as well as the promotion of sustainable development of China’s dry lands [4].

The Gonghe Basin of the Tibet Plateau is severely affected by desertification, with a desertification area of 1.27×10^4^ km^2^, which accounts for 91.9% area of the basin [5]. *Caragana intermedia* Kuang et H. C. Fu [6, 7], a perennial leguminous shrub, has been widely planted after the erection of straw checkerboards on shifting sand dunes in this region. Legume shrub species may be a critical regulator of soil nutrient dynamics because of their high foliar nitrogen and potential for symbiotic nitrogen fixation [8]. Previous studies have shown that the existed of leguminous shrub resulted in spatial heterogeneity of soil properties. For instance, some studies have shown that after the establishment of *Caragana microphylla* plantation on sand dunes, soil nutrient concentrations were higher under the shrubs than outside the canopies [9–10]. *Retama sphaerocarpa* is a leguminous shrub found in most regions of Spain, frequently with a growth of herbs in its understorey which increased the production of litter, and the interception of wind-blown dust, forming so-called “islands of fertility” [11–13]. The studies of *Caragana tibetica* nebkhas on the Inner Mongolia Plateau have shown that the “fertile islands” are formed inside and underneath *C*. *tibetica* nebkhas [14]. Formation of “fertile islands” increases spatial heterogeneity in soil resources [15], and affects not only seedling establishment [16], but also the spatial distribution of plant productivity and diversity [17–18].

All of the above studies analyzed the spatial distribution of soil nutrients in other leguminous shrub species in other regions. However, very few studies have evaluated the spatial heterogeneity of soil nutrients in *C*. *intermedia* shrub land, especially for alpine sandy land of the Tibet Plateau, with very high elevation and cold climate, lack of organic matter, nutrient and water. In this harsh environment, analysis the spatial distribution of soil nutrients and its impact factors are even more important, it will be helpful for understanding the ecological relationship between soil and environment [19]. Therefore, we performed a sampling campaign in *C*. *intermedia* plantation on sand dunes to test two general hypotheses that: (1) spatial heterogeneity of soil nutrients exists in the *C*. *intermedia* plantation on sand dunes of Gonghe Basin; (2) “fertile islands” are formed after establishment of *C*. *intermedia* plantations in alpine sandy land.

## Materials and Methods

### Study Area

This study was conducted in the desertification combating experimental site of the Qinghai Gonghe Desert Ecosystem Research Station (99°45′–100°30′E, 36°03′–36°40′N and altitude 2871 m). The station is located in the Gonghe Basin of the northeastern Tibet Plateau. This station is a part of the Chinese Desert Ecosystem Research Network (CDERN) of the State Forestry Administration of P.R. China. The station was constructed by the Chinese Academy of Forestry and the Desertification Combating Station of Qinghai Province. No specific permissions were required to conduct research in this study site, and the field studies did not involve endangered or protected species. The climate in the study area is a plateau continental climate with a mean annual temperature of 2.4°C, mean annual precipitation of 246.3 mm and mean annual potential evaporation of 1716.7 mm. The mean annual frost-free period is only 91 days, and the total solar radiation is 6631.69 MJ·m^-2^·y^-1^. The mean annual number of windy days is 50.6 days (up to 97 days), and the mean annual number of sandstorm days is 20.7 days. The main wind directions are west and northwest, with a mean annual wind speed of 2.7 m·s^-1^; the maximum wind speed reaches 40 m·s^-1^. The zonal soils are chestnut soil and brown soil, while the azonal soils are aeolian, meadow and bog soils. In the research area, the main vegetation type is sand-fixing plantation, including tree species, e.g., *Populus cathayana* Rehd and *Populus simonii* Carr, and shrub species, e.g., *C*. *intermedia*, *C*. *korshinskii* Kom., *Hippophae rhamnoides* L., *Salix cheilophila* Schneid., and *Salix psammophila* C. Wang et Chang Y. Yang [7].

*Caragana intermedia* is the main species used for vegetation restoration on sand dunes in Gonghe Basin. Before *C*. *intermedia* were planted, straw checkerboards (1 m × 1 m) were established on shifting sand dunes. This study was conducted on four fixed sand dunes, running in a northeast—southwest direction, on which *C*. *intermedia* was planted in May 2000. The belts of *C*. *intermedia* run parallel to the direction of the sand dunes, with 2.5 m between each row and 1 m between plants in each row. The mean length, width and height of sand dunes were approximately 90, 50 and 6 m, respectively. The morphological characteristics of 10 *C*. *intermedia* plants were measured at each dune position; these characteristics included height (height of highest shoot), crown diameter (maximum diameter of the shrub canopy from Northeast—Southwest and Southeast—Northeast) and ground diameter (maximum diameter of the shrub shoots from Northeast—Southwest and Southeast—Northeast at ground level). The morphological characteristics of *C*. *intermedia* on the different slopes are shown in Table 1.

**Table 1:** Morphological characteristics of *C*. *intermedia* plantation on different slopes on sand dunes.

Slopes	Height (cm)	Crown diameter (cm)		Ground diameter (cm)	
		Northeast—Southwest	Southeast—Northwest	Northeast—Southwest	Southeast—Northwest
**Windward slopes**	121.90±6.00a	105.60±6.88a	152.20±7.09a	24.80±1.70b	37.40±2.52b
**Dune tops**	117.00±8.62a	110.50±9.89a	153.60±6.79a	22.00±1.29ab	28.50±0.86a
**Leeward slopes**	117.80±3.26a	104.40±10.33a	140.90±5.50a	19.70±0.83a	24.40±0.78a

Values are means of ten measurements ± standard error. Different lowercase letters following values indicate a significant difference in morphological characteristics on different slopes, according to Duncan’s multiple range test (*P*<0.05).

### Experiment Design and Soil Sampling

Field work was conducted in June 2012. Three different topographical positions were selected on four sand dunes covered by *C*. *intermedia*: the middle of windward slopes, the middle of leeward slopes and dune tops. On each sand dune, four lines of *C*. *intermedia* were randomly selected as sampling sites. Soil samples were collected under shrubs and between shrubs with the depth of 0–5, 5–10, 10–20, 20–30 and 30–50 cm. Four soil samples collected under shrubs from four *C*. *intermedia* lines on the same slope at the same depth were mixed into one soil sample; similarly, four soil samples collected between shrubs from four *C*. *intermedia* lines on the same slope at the same depth were mixed into one soil sample, and four sand dunes as four repetitions. The sampling location under shrubs was located 30 cm from the center of the shrub; the sampling location between shrubs was in the middle of two *C*. *intermedia* lines. The samples at 0–5 cm depth were collected by shovel, while deeper samples were collected using a 10-cm-diameter AMS soil auger. Soil samples were placed in zip-lock bags and air-dried for soil nutrient measurements. A total of 120 soil samples were collected on sand dunes (3 positions × 2 microsites × 5 soil depths × 4 replicates).

### Vegetation sampling

Vegetation investigation was carried out in August 2012. Ten 1×1 m^2^ quadrats were established randomly between shrubs for each slope position. At each selected quadrate, we assessed the number of herbaceous plant species and the height of each species. The herbaceous vegetation cover was estimated using the quadrats. Annual and perennial herbaceous vegetation increased after *C*. *intermedia* plantation establishment, the main species being *Suaeda glauca* (Bunge) Bunge, *Leymus secalinus* (Georgi) Tzvel and *Artemisia sieversiana* Ehrhart ex Willd. The nomenclature of main species included in this work follows Flora [6] and Flora Qinghaiica [20].

### Soil Laboratory Analysis

Samples for soil nutrient measurement were hand-sieved through a 20—mesh sieve to remove roots and other debris. Half of each sample was put back into the zip-lock bag for available nutrient analyses, and the other half was sieved through a 100—mesh sieve for total nutrient and soil organic matter (SOM) analyses. All analyses were based on Physical and Chemical Analysis Methods of Soils [21]. SOM was measured by the K_2_Cr_2_O_7_–H_2_SO_4_ oxidation method. Total nitrogen (TN) and available nitrogen (AN) were measured by the Kjeldahl method. Total phosphorus (TP) was measured by the Mo-Sb colorimetric method, available phosphorus (AP) by the NaHCO_3_ extraction—Mo-Sb colorimetric method. Total potassium (TK) was measured by atomic absorption, whereas available potassium (AK) was measured by the ammonium acetate extraction atomic absorption method.

### Statistical Analysis

All results are reported as the mean (four measurements) ± standard error (SE). One-way ANOVA was used to compare morphological characteristics of *C*. *intermedia* among slopes, soil nutrient contents among treatments, and Duncan’s multiple range tests was used to evaluate differences among the means. The *t*-test was used to show enrichment effects (the difference between under shrub and between shrub measurements). Correlation Analysis was used to analysis the correlation among soil nutrient indicators (at 0–5 cm), shrubs morphological characteristics and herbaceous coverage. SPSS 16.0 software was used for the above statistical analyses. The threshold of statistical significance was set at *P*<0.05 for all analyses. To identify differences in the soil nutrient contents under the shrub canopy (A) and between the shrub canopy (B), the enrichment ratio (E) was determined, where E = A/B [9, 22]. Redundancy Analysis (RDA) was used to evaluate the relationship between soil nutrients and environmental factors. We have two sets of variables, response variables and explanatory variables. The response variables are soil nutrients, and they are quantitative variables. The explanatory variables fall within two categories: quantitative variables (soil depth), and categorical variables (topography and microsite). All raw data were standardized (y = log (x + 1)), the significant of variables were tested 999 times by Monte Carlo, CANOCO 4.5 software was used for this statistical analyses.

## Results

### The Distribution of SOM, TN, TP, TK, AN, AP and AK in *C*. *intermedia* Plantations on Sand Dunes

The SOM content under shrubs for windward slopes and between shrubs for all slopes was highest at 0–5 cm and significantly decreased with greater soil depth (*P*<0.05) (Table 2). The TN, AN and AP content under and between shrubs for all slopes at the 0–5 cm depth was significantly higher than at other depths (*P*<0.05).

**Table 2:** Spatial distribution of soil nutrients in *C*. *intermedia* plantation on sand dunes (mean ± SE).

Index	Soil depth	Under shrubs			Between shrubs		
	(cm)	Windward slopes	Dune tops	Leeward slopes	Windward slopes	Dune tops	Leeward slopes
**SOM (g/kg)**	0–5	3.49±0.09Aa	2.29±0.06Ba	2.83±0.13Ba	3.38±0.09Aa	2.83±0.09Ba	2.76±0.18Ba
	5–10	3.04±0.17Aab	2.85±0.08Aa	2.58±0.25Aa	2.80±0.23Ab	2.56±0.11Aab	2.46±0.21Aa
	10–20	2.62±0.12Abc	2.80±0.10Aa	2.48±0.25Aa	2.36±0.09Abc	2.30±0.23Aabc	2.31±0.21Ab
	20–30	2.19±0.22Ac	2.61±0.10Aa	2.19±0.17Aa	1.96±0.20Acd	2.00±0.27Abc	1.83±0.20Abc
	30–50	1.66±0.17Ad	2.23±0.31Aa	2.02±0.18Aa	1.57±0.13Ad	1.84±0.27Ac	1.44±0.05Ac
**TN (g/kg)**	0–5	0.55±0.02Aa	0.40±0.03Ba	0.43±0.03Ba	0.39±0.02Aa	0.35±0.02Aa	0.40±0.03Aa
	5–10	0.40±0.04Ab	0.27±0.03Bb	0.28±0.03Bb	0.30±0.03Ab	0.23±0.03Ab	0.22±0.01Ab
	10–20	0.37±0.03Abc	0.24±0.01Bb	0.27±0.03Bb	0.27±0.02Ab	0.21±0.02Ab	0.22±0.02Ab
	20–30	0.33±0.01Abc	0.23±0.02Bb	0.25±0.02Bb	0.27±0.02Ab	0.21±0.02Bb	0.19±0.01Bb
	30–50	0.32±0.01Ac	0.22±0.01Bb	0.25±0.02Bb	0.26±0.01Ab	0.18±0.01Bb	0.19±0.01Bb
**TP (g/kg)**	0–5	0.44±0.03Aa	0.23±0.02Ba	0.33±0.05Ba	0.33±0.04Aa	0.21±0.01Ba	0.25±0.01Ba
	5–10	0.36±0.03Aab	0.24±0.01Ba	0.28±0.03Ba	0.30±0.03Aa	0.20±0.01Ba	0.23±0.02Ba
	10–20	0.35±0.04Aab	0.22±0.02Ba	0.27±0.03ABa	0.32±0.01Aa	0.21±0.01Ba	0.22±0.03Ba
	20–30	0.32±0.01Abc	0.24±0.03Ba	0.23±0.02Ba	0.30±0.02Aa	0.21±0.02Aa	0.22±0.03Aa
	30–50	0.25±0.01Ac	0.22±0.02Ba	0.21±0.01Aa	0.25±0.04Aa	0.20±0.01Aa	0.23±0.02Aa
**TK (g/kg)**	0–5	14.82±0.16Aa	12.65±0.71Ba	13.75±0.10ABa	13.25±0.33Aa	11.84±0.39Ba	12.70±0.25ABa
	5–10	14.57±0.12Aa	12.59±0.63Ba	12.68±0.13Ba	11.54±1.19Aa	10.54±0.84Aa	11.33±0.89Aa
	10–20	14.76±0.09Aa	12.46±0.37Ba	11.92±0.65Ba	11.30±1.17Aa	11.30±0.56Aa	12.38±0.44Aa
	20–30	14.70±0.12Aa	11.55±0.64Ba	12.21±0.24Ba	12.19±0.32Aa	12.57±0.46Aa	12.88±0.52Aa
	30–50	14.63±0.25Aa	11.83±0.36Ba	12.38±0.69Ba	12.31±0.56Aa	12.08±0.50Aa	13.09±0.05Aa
**AN (mg/kg)**	0–5	114.59±1.47Aa	106.05±4.08Aa	108.12±3.35Aa	95.45±4.08Aa	77.77±4.08Ba	79.22±3.45Ba
	5–10	98.98±5.77Aa	91.91±4.08Aa	91.91±4.08Aab	88.38±3.54Aa	40.12±13.66Bb	57.79±7.21Bb
	10–20	72.60±10.07Ab	65.46±8.78Ab	71.49±12.11Abc	74.24±3.54Ab	52.17±9.03Bb	57.57±4.64ABb
	20–30	62.04±9.84Ab	60.73±6.98Ab	65.75±3.21Ac	74.24±3.54Ab	48.28±8.95Ab	61.57±5.54Ab
	30–50	56.08±9.86Ab	56.70±9.98Ab	50.63±7.56Ac	70.70±5.77Ab	42.38±8.35Bb	62.86±3.36Ab
**AP (mg/kg)**	0–5	5.29±0.15Aa	4.53±0.15Ba	4.32±0.15Ba	4.61±0.16Aa	3.93±0.11Ba	3.93±0.15Ba
	5–10	3.95±0.07Ab	3.30±0.15Bb	3.55±0.10Bb	3.72±0.07Ab	3.08±0.04Bb	2.91±0.11Bb
	10–20	3.68±0.04Ab	2.91±0.13Cbc	3.30±0.11Bb	3.64±0.08Ab	2.87±0.21Bb	2.79±0.11Bb
	20–30	3.72±0.14Ab	3.00±0.16Bbc	3.21±0.12Bb	3.51±0.08Ab	2.70±0.07Bb	2.83±0.23Bb
	30–50	3.72±0.12Ab	2.83±0.13Cc	3.30±0.05Bb	3.64±0.05Ab	2.74±0.15Bb	2.91±0.11Bb
**AK (mg/kg)**	0–5	104.69±0.82Aa	102.23±0.53Aa	103.84±0.60Aa	95.69±1.03Aa	94.66±4.95Aa	94.13±2.94Aa
	5–10	100.31±1.81Ab	98.26±2.37Aa	99.94±1.44Aab	91.98±1.67Aa	92.44±3.56Aa	92.99±1.31Aa
	10–20	91.64±1.13Ac	96.51±3.94Aa	94.74±2.50Abc	90.72±2.43Aa	89.97±1.80Aa	91.16±1.21Aab
	20–30	89.17±1.59Acd	89.79±0.38Ab	89.52±2.53Acd	90.78±2.27Aa	87.84±1.29Aa	86.76±1.66Abc
	30–50	86.88±0.91Ad	89.05±0.77Ab	87.51±1.52Ad	88.05±1.07Aa	88.07±0.67Aa	84.69±1.07Ac

Notes: Different uppercase letters following values indicate a significant difference in soil nutrient contents at different dune positions; different lowercase letters following values indicate a significant difference in soil nutrient contents at different soil depths, according to Duncan’s multiple range test (P<0.05), n = 4.

The SOM content under and between shrubs at 0–5 cm depth was significantly higher in windward slopes than in dune tops and leeward slopes (*P*<0.05). The TN content under shrubs at all soil depths, AN content between shrubs at the 0–5 and 5–10 cm depths was significantly higher in windward slopes than in dune tops and leeward slopes (*P*<0.05). The TP content under and between shrubs at 0–5 and 5–10 cm depth, AP content at all depths was significantly higher in windward slopes than in dune tops and leeward slopes (*P*<0.05). The TK content under and between shrubs at 0–5 cm was significantly higher in windward slopes than in dune tops (*P*<0.05).

### Effects of Shrubs on Soil Nutrients Enrichment for Different Slopes

The *t*-test analysis showed that on windward slopes, the levels of TN, TP and TK were significantly higher under shrubs than between shrubs (*P*<0.05) (Table 3). On dune tops, SOM, TP, AN and AK levels were significantly higher under shrubs than between shrubs (*P*<0.05). On leeward slopes, levels of AN, AP and AK were significantly higher under shrubs than between shrubs (*P*<0.05). For the contents of SOM, TN, TP, TK, AP and AK of all slopes, E was> 1.00. The E for AN content on dune tops and leeward slopes was also > 1.00. The E of TN content on windward (1.33) and leeward slopes (1.26) was higher than for other nutrients. The E of AN content on dune tops (1.62) was higher than for other nutrients.

**Table 3:** Enrichment ratio (E) of SOM (soil organic matter), TN (total nitrogen), TP (total phosphorus), TK (total potassium), AN (available nitrogen), AP (available phosphorus) and AK (available potassium) at different dune positions.

Variation		Windward slopes		Dune tops		Leeward slopes	
		US	BS	US	BS	US	BS
**SOM (g/kg)**	Mean	2.60±0.16a	2.42±0.16a	2.68±0.09a	2.30±0.0.12b	2.42±0.10a	2.16±0.13a
	E	1.09		1.25		1.16	
**TN (g/kg)**	Mean	0.39±0.02a	0.30±0.01b	0.27±0.02a	0.23±0.02a	0.30±0.02a	0.24±0.02a
	E	1.33		1.33		1.26	
**TP (g/kg)**	Mean	0.34±0.02a	0.30±0.01b	0.23±0.01a	0.21±0.01b	0.27±0.01a	0.23±0.01a
	E	1.19		1.13		1.19	
**TK (g/kg)**	Mean	14.70±0.07a	12.52±0.42b	12.22±0.29a	12.67±0.30a	12.59±0.18a	12.47±0.23a
	E	1.20		1.06		1.01	
**AN (mg/kg)**	Mean	80.85±6.07a	80.60±2.73a	76.17±5.28a	52.14±4.31b	77.58±5.40a	63.80±2.67b
	E	0.99		1.62		1.25	
**AP (mg/kg)**	Mean	4.07±0.15a	3.82±0.10a	3.31±0.15a	3.07±0.12a	3.53±0.10a	3.07±0.12b
	E	1.05		1.07		1.16	
**AK (mg/kg)**	Mean	95.24±1.92a	91.44±0.91a	95.17±1.42a	90.60±1.31b	95.11±1.59a	89.95±1.09b
	E	1.03		1.05		1.06	

Notes: US, under shrubs; BS, between shrubs. The *t*-test was used to show enrichment effect (the difference between US and BS). Different lowercase letters following values indicate a significant difference in SOM, TN, TP, TK, AN, AP and AK under and between shrubs, according to *t*-test (*P*<0.05).

### Relationship between soil nutrients and environmental factors

**Fig 1:**
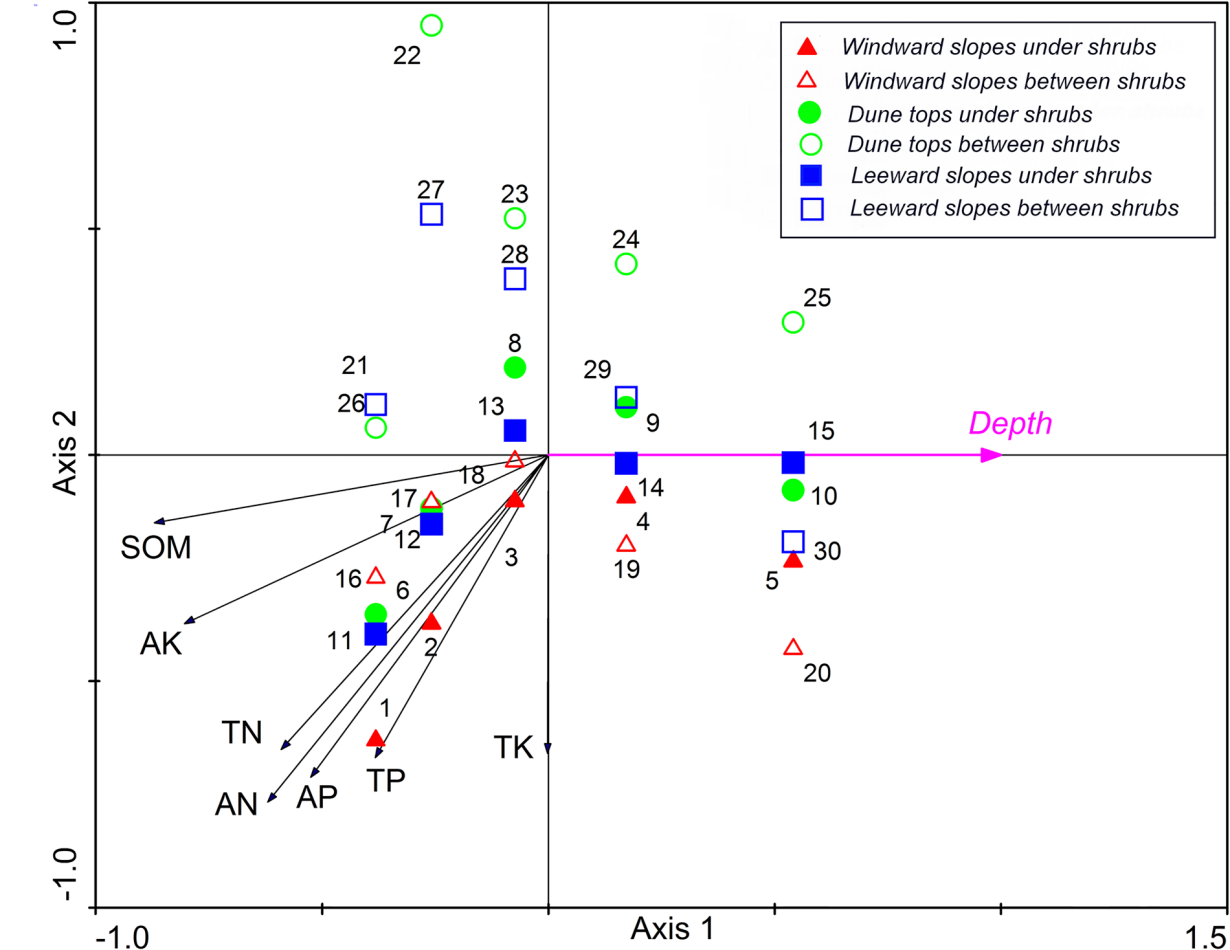
RDA two-dimensional ordination diagram of the first two axes showing the correlation of soil nutrients and environmental factors.

Redundancy analysis (RDA) visualizes correlations of soil nutrients with environmental factors (Fig 1). A total of 86.7% of the cumulative variance of the soil-environment relationship was represented by the first two axes. This result indicated a strong association between soil nutrients and the measured environmental factors. Samples in the positive part of axis 1 had higher depths, those in the negative part were located in the upper soil layers. Therefore, axis 1 represented soil depth, and it was the most important driver of soil nutrients in the study. Axis 2 was in the same direction with topography (with the direction of windward slope—leeward slope—dune top) and microsites (with the direction of under shrubs to between shrubs), therefore, axis 2 represented topography and microsites. Soil nutrients increased towards the negative part of axis 1 and 2, suggesting that nutrients increased in the upper soil, windward slope and under shrubs.

There was a convergence towards more soil nutrients in all topography positions and microsites at shallower soil layers. Windward slopes with the highest soil nutrients level, followed by leeward slopes, the dune tops with the lowest soil nutrients level. The difference of soil nutrients level between topography positions was more marked in between shrubs. In the dune tops, the soil nutrients level under shrubs was higher than between shrubs. The difference of soil nutrients level between “under shrubs” and “between shrubs” for dune tops was more marked than other topography positions. In the leeward slopes, the soil nutrients level under shrubs was higher than between shrubs except for 30–50 cm depth. Contrary to the other topography positions, windward slopes had less difference of soil nutrients level between “under shrubs” and “between shrubs”. All of these mean that “fertile islands” were formed, especially for shallow soil layers, and the “fertile islands” were more marked on lower soil nutrients level topography positions, while it decreased towards higher soil nutrients level topography positions.

## Discussion

Low levels of soil nutrients are a major feature in arid and semi-arid ecosystems. The fixation of shifting sand dunes lies primarily in the restoration of vegetation and improvement of soil fertility [23]. The shrubs are the main species and they are very important in the ecological function of arid and semi-arid ecosystems. Some shrub species play crucial roles in preventing desertification and in restoration of degraded arid lands [24]. At the same time, the presence of shrubs strongly influences soil nutrients distribution [25].

This study showed that spatial heterogeneity of soil nutrients was existed in *C*. *intermedia* plantation on sand dunes. Depth was the most important environment factor influencing soil nutrient distribution on sand dunes (Fig 1), more soil nutrients (SOC, TN, TP, AN, AP and AK) accumulated on surface soil (0–5 cm) and decreased with soil depth (Table 2), indicating that soil nutrients had undergone surface aggregation. The possible reason for this phenomenon is that litter and windblown fine materials were trapped by shrubs and accumulated on surface soil [10], their nutrients returned to soil via microbial decomposition [22], which leads to higher nutrient level. The studies about *C*. *microphylla* plantation on sand dunes have also shown that soil nutrients were higher on surface soil than in deeper layer soil [9].

Topography is an important factor to consider in the process of vegetation restoration, as it affects soil moisture, soil temperature and air flow, there by affecting the spatial heterogeneity of soil properties [26–28]. Our study showed that more soil nutrients accumulated on windward slopes, especially on surface soil (Table 2). To analyze this phenomenon, we did a correlation analysis among surface soil nutrients, shrubs morphological characteristics and herbaceous coverage (Table 4). The herbaceous coverage was significantly positive correlated with SOM, TP, AP (*P*<0.01), TN, TK (*P*<0.05). The coverage of herbaceous vegetation for windward slopes was 30.3%, much higher than dune tops (14.7%) and leeward slopes (18.8%) (Table 5). The development of herbaceous plants on sand dunes was an important part of net primary productivity, and the plants’ rapid growth and death were important ways by which soil nutrients accumulated [29–30]. Therefore, the higher coverage of herbaceous vegetation is a possible reason for more soil nutrients accumulated on windward slopes. The ground diameter of *C*. *intermedia* was also significantly positive correlated with SOM, TN, TK (*P*<0.01), AN, AP (*P*<0.05) (Table 4). The ground diameter of *C*. *intermedia* on windward slopes was larger than on dune tops and leeward slopes (Table 1). Larger ground diameters may be related to its larger root system, which results in a high level of nitrogen fixation and even more soil microbes, and it could be conductive to the soil amelioration. Other studies also showed that the sediment amount and fine materials were positively and significantly correlated with shrubs ground shoots number and shoots diameter [31]. In addition, in alpine sandy lands with a semi-arid climate, wind erosion was changed by topography and plantation. Wind speed was reduced when it went through the plantations [23], maybe more wind erosion materials and litter were trapped and concentrated under the shrubs when sand flow went through windward slopes. When the impaired sand flow reached the dune tops and leeward slopes, less wind erosion materials, dust and litter were trapped by *C*. *intermedia*, and this may be another reason for more soil nutrients distributed on windward slopes, however, further study is needed for clarification.

**Table 4:** Pearson’s correlation among soil nutrient indicators (at 0–5 cm), morphological characteristics of shrub and herbaceous coverage.

Parameters	SOM	TN	TP	TK	AN	AP	AK	H	C1	C2	G1	G2	H-C
**SOM**	1.00												
**TN**	0.77**	1.00											
**TP**	0.44	0.62*	1.00										
**TK**	0.52	0.48	0.58*	1.00									
**AN**	0.49	0.66*	0.38	0.68*	1.00								
**AP**	0.80**	0.59*	0.43	0.48	0.44	1.00							
**AK**	0.41	0.72**	0.48	0.60*	0.49	0.45	1.00						
**H**	0.178	0.132	-0.11	0.38	0.55	-0.20	-0.03	1.00					
**C1**	-0.28	-0.31	-0.38	-0.20	-0.21	-0.46	-0.26	0.39	1.00				
**C2**	-0.02	-0.04	-0.17	-0.33	-0.01	-0.27	-0.48	0.26	0.19	1.00			
**G1**	0.57	0.48	0.42	0.76**	0.64*	0.53	0.38	0.46	0.23	-0.14	1.00		
**G2**	0.81**	0.73**	0.53	0.46	0.48	0.70*	0.40	0.18	-0.10	-0.24	0.67*	1.00	
**H-C**	0.83**	0.69*	0.71**	0.67*	0.39	0.79**	0.51	-0.09	-0.39	-0.14	0.55	0.72**	1.00

H, shrubs height; C1, shrubs crown diameter with the direction of Northeast—Southwest; C2, shrubs crown diameter with the direction of Southeast—Northwest; G1, shrubs ground diameter with the direction of Northeast—Southwest; G2, shrubs ground diameter with the direction of Southeast—Northwest; H-C, herbaceous coverage.

*Correlation significant at the 0.05 level (2-tailed).

**Correlation significant at the 0.01 level (2-tailed).

**Table 5:** Distribution of herbaceous in *C*. *intermedia* plantation on sand dunes.

Species name	Life form	Windward slopes			Dune tops			Leeward slopes		
		N	H (cm)	F%	N	H (cm)	F%	N	H (cm)	F%
***Suaeda glauca* (Bunge) Bunge**	AF	49.8	8	90	107	7.9	100	39.7	9	80
***Leymus secalinus* (Georgi) Tzvel.**	PG	75.2	46.4	100	12.6	29.6	70	58.2	47.2	100
***Artemisia sieversiana* Ehrhart ex Willd.**	AF	54.4	25.1	80	2.7	18.7	30	33.7	22.7	70
***Salsola collina* A. J. Li**	AF	6.8	8.6	80	5.4	10.2	60	13.8	11.4	50
***Artemisia desertorum* Spreng.**	PS	2.9	32.2	50	1.3	31.2	50	0.1	36	10
***Plantago minuta* Pall.**	AF	1.2	8.7	30	1.7	5.7	60	0.1	8	10
***Lappula myosotis* v. Wolf, Gen**	AF	0.9	11.7	30	0.8	11.5	20	4.8	18	40
***Heteropappus altaicus* (Willd.) Novopokr.**	PF	0.8	37.5	40	0.4	23.7	30			
***Glycyrrhiza uralensis* Fisch.**	PL	0.2	32	10						
***Sonchus oleraceus* Linn.**	PF							1.5	34.5	20
***Convolvulus arvensis* Linn.**	PF	0.9	17	10						
***Lepidium apetalum* Willd**	AF	0.2	16	10						
***Setaria viridis* (Linn.) Beauv.**	AG				0.7	8	10	0.1	6	10
***Achnatherum splendens* (Trin.) Nevski**	PG							0.1	119	10
***Peganum multisectum*) (Maxiam.) Bobr.**	PF							0.1	28	10
**Total number of individuals**		193.3			132.6			152.2		
**Total number of species**		11			9			11		
**Coverage (%)**		30.3			14.7			18.8		

N, numbers of individuals; H, height of species; F%, frequency; AF, annual forbs; AG, annual grass; PF, perennial forbs; PG, perennial grass; PL, perennial legume; PS, perennial shrub.

More soil nutrients concentrated under shrubs than between shrubs on sand dunes (Table 3), soil nutrients were obviously enriched by *C*. *intermedia*, and a typical “fertile islands” were formed in this semi-arid region, especially for shallow soil layers. This is similar to other plantations in Horqin Sandy Land and Loess Plateau [22, 32]. There are several possible reasons for the formation of “fertile islands” in *C*. *intermedia* plantations. One of the possible reasons is that shrubs reduce wind velocity and soil erosion [31], trap windblown fine materials; and thus deposition of dust concentrates nutrients below their canopy [33], which creates “fertile islands” in a low-nutrient matrix [34]. Another possible reason is that rainfall containing atmospheric dust was intercepted by the canopy, and the nutrients in the dust were combined with leaf secretions, leading to an increase in soil nutrients [35]. In addition, root exudates and the deposition of dead roots in the rhizosphere may increase soil nutrient levels. For example, SOM, TN, AN and AP contents of *C*. *microphylla* and *Artemisia halodendron* were all higher in the rhizosphere than non-rhizosphere soil in Horqin Sandy Land [36]. The E ratio for TN on windward and leeward slopes, as well as the E ratio for AN on dune tops, was higher than for other nutrients, most likely because *C*. *intermedia* is a leguminous shrub and has a large number of rhizobia with high nitrogenase activity in the root systems, which can fix free nitrogen in the air [32]. Similarly, the E for TN was higher than that for TP and TK in *C*. *microphylla* in Horqin Sandy Land [9].

## Conclusions

The results of this study showed that more soil nutrients accumulated on surface soil (0–5 cm) and decreased with greater soil depth, more soil nutrients distributed on windward slopes, and more soil nutrients concentrated under shrubs. These results indicate that spatial heterogeneity of soil nutrients was existed in *C*. *intermedia* plantation on sand dunes in Gonghe Basin, and soil “fertile islands” were formed under shrubs, especially for shallow soil layers. Understanding the spatial heterogeneity of soil nutrients in *C*. *intermedia* plantation on sand dunes was significant for the establishment and management of shrub plantations in alpine sandy land.
