# Correction: Spatial Heterogeneity of Soil Nutrients after the Establishment of *Caragana intermedia* Plantation on Sand Dunes in Alpine Sandy Land of the Tibet Plateau

**DOI:** 10.1371/journal.pone.0218503

**Published:** 2019-06-12

**Authors:** Qingxue Li, Zhiqing Jia, Yajuan Zhu, Yongsheng Wang, Hong Li, Defu Yang, Xuebin Zhao

Following publication of this article [1], concerns were raised about partial overlap with a previously published Chinese-language article by some of the same authors in the journal *Forest Research* [2], which is closely related, and should have been cited and discussed.

The authors clarify that the *PLOS ONE* study is based on the same *C*. *intermedia* plantation previously reported in the *Forest Research* article, and therefore, some of the data have previously been reported, including:

- The data on the distribution and morphological characteristics of *C*. *intermedia* on different slopes of the sand dunes (Table 1 and Table 5 of the *PLOS ONE* article). Note that the morphological characteristics of *C*. *intermedia* in the *PLOS ONE* article were calculated from 10 plants, and the morphological characteristics of *C*. *intermedia* in the *Forest Research* article were calculated from 20 plants; therefore, some of the data are slightly different.
- Measurements of SOM, TN, TP and TK at different soil depths and in different positions. The data on SOM and TP content between shrubs (Table 2 of the *PLOS ONE* article) was re-used from the previous study [2]. The authors acknowledge some differences between the SOM between shrubs data for leeward slopes at 0-5cm depth reported for the two articles and confirm that the *PLOS ONE* data are correct.

Both articles examined the spatial variation of soil nutrients after the establishment of *C*. *intermedia* in relation to the slope aspect. The previous study about the effects of sand dune slopes on soil nutrients and plant community for *C*. *intermedia* plantation [2] found that the SOM, TN and TP content for all sand dunes slopes of *C*. *intermedia* plantation were significantly higher than the moving sand dunes; the SOM, TN and TP content in shallow soil for windward slopes were significantly higher than that at the top of slopes and leeward slopes; and the coverage and species of herbaceous increased, especially for windward slopes [2]. Based on the previous study [2], a more systematic study was carried out on the spatial heterogeneity of soil nutrients after *C*. *intermedia* plantation was established on sand dunes. The *PLOS ONE* article included novel analyses of the correlation between soil nutrient levels and environmental factors, and data from SOM, TN, TP and TK under shrubs, TN and TK between shrubs and available soil nutrients were also newly reported in the *PLOS ONE* article.

There are errors in the numbering of references 9–14 of the original article [1]. The corrected numbering and order of these references is as follows:

9. Dong XW, Zhang XK, Bao XL, Wang JK. Spatial distribution of soil nutrients after the establishment of sand-fixing shrubs on sand dune. Plant Soil Environ. 2009; 55(7): 288–294.

10. Cao CY, Jiang SY, Zhang Y, Zhang FX, Han XS. Spatial variability of soil nutrients and microbiological properties after the establishment of leguminous shrub *Caragana microphylla* Lam. plantation on sand dune in the Horqin Sandy Land of Northeast China. Ecol Eng. 2011; 37(10): 1467–1475.

11. Pugnaire FI, Haase P, Puigdefábregas J, Cueto M, Clark SC, Incoll LD. Facilitation and succession under the canopy of a leguminous shrub, *Retama sphaerocarpa*, in a semi-arid environment in south-east Spain. OIKOS. 1996; 76: 455–464.

12. Pugnaire FI, Armas C, Valladares F. Soil as a mediator in plant-plant interactions in a semi-arid community. J VEG SCI. 2004; 15: 85–92.

13. Moro MJ, Pugnaire FI, Haase P, Puigdefábregas J. Effect of the canopy of *Retama sphaerocarpa* on its understory in a semiarid environment. Funct Ecol. 1997; 11: 425–431.

14. Zhang PJ, Yang J, Zhao LQ, Bao SRL, Song BY. Effect of Caragana tibetica nebkhas on sand entrapment and fertile islands in steppe-desert ecotones on the Inner Mongolia Plateau, China. Plant Soil. 2011; 347(1–2): 79–90.

Additionally, the Data Availability statement for this paper [1] is incorrect. The relevant data are not provided within the paper and its Supporting Information files. The full underlying data set was provided by the authors at the time of submission; however, the Supporting Information file was not published. The authors have provided the underlying data set here as Supporting Information file S1 Dataset.

## Supporting information

S1 Dataset Supplementary dataset. (XLS)
